# Supplementary material for: Moisture alone is sufficient to impart strength but not weathering resistance to termite mound soil
Source: R Soc Open Sci. 2020 Aug 12;7(8):200485. doi: 10.1098/rsos.200485 (PMC7481702; doi:10.1098/rsos.200485)
Supplement: Soil processing images and tests for peak compressive strength [file rsos200485supp1.pdf]

Supplementary

Moisture alone is sufficient to impart strength but not weathering resistance to termite mound soil

Nikita Zachariah[1], Tejas G. Murthy[2], Renee M. Borges[1 *]

[1]Centre for Ecological Sciences, Indian Institute of Science, Bangalore 560012, India

[2]Department of Civil Engineering, Indian Institute of Science, Bangalore 560012, India

* Corresponding author; Email: renee@iisc.ac.in; Phone: +91-80-23602972;

ORCID: 0000-0001-8586-7380

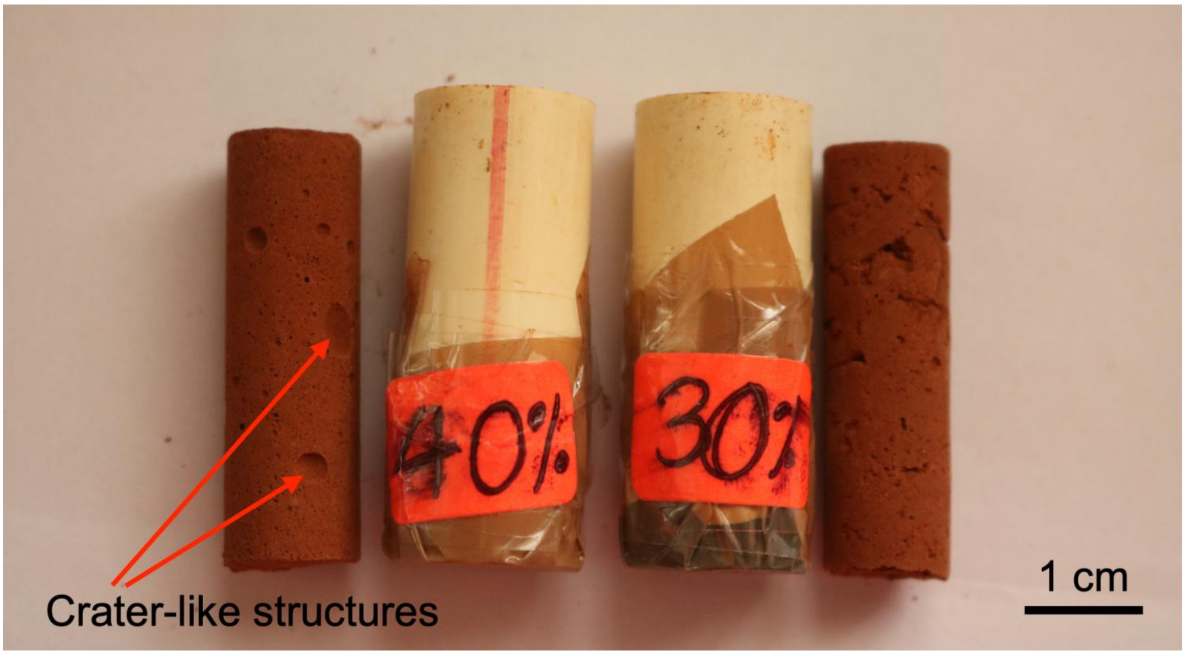

Figure S1. Moulds used for sample preparation for testing soil strength. Moulds were sealed from the base with adhesive tape and and allowed drying only from the top. Crater-like structures are visible in the samples moulded at 40% moisture content.

Table S1. Tukey's HSD post-hoc test for peak compressive strength of soil under different days of drying ($\alpha = 0.05$). In column ‘Comparison’ the number on the right is compared to the number on the left. A positive sign in the ‘Mean diff.’ column suggests that the number to the left in the ‘Comparison’ column is greater than the number on the right.

Comparison	Mean diff.	95% CI of diff.	Summary
10 days vs. 9 days	32.061	-483.34 to 547.46	>0.05
11 days vs. 9 days	-44.829	-585.38 to 495.72	>0.05
12 days vs. 9 days	556.317	15.75 to 1096.87	<0.05
13 days vs. 9 days	286.471	-254.08 to 827.02	>0.05
14 days vs. 9 days	89.105	-451.45 to 629.66	>0.05
16 days vs. 9 days	398.871	-116.53 to 914.27	>0.05
11 days vs. 10 days	-76.890	-617.44 to 463.66	>0.05
12 days vs. 10 days	524.255	-16.30 to 1064.81	>0.05
13 days vs. 10 days	254.410	-286.14 to 794.96	>0.05
14 days vs. 10 days	57.044	-483.51 to 597.60	>0.05
16 days vs. 10 days	366.810	-148.59 to 882.21	>0.05
12 days vs. 11 days	601.146	36.55 to 1165.74	<0.05
13 days vs. 11 days	331.300	-233.29 to 895.89	>0.05
14 days vs. 11 days	133.934	-430.66 to 698.52	>0.05
16 days vs. 11 days	443.700	-96.85 to 984.25	>0.05
13 days vs. 12 days	-269.845	-834.44 to 294.74	>0.05
14 days vs. 12 days	-467.211	-1031.80 to 97.38	>0.05
16 days vs. 12 days	-157.445	-698.00 to 383.11	>0.05
14 days vs. 13 days	-197.365	-761.96 to 367.22	>0.05
16 days vs. 13 days	112.400	-428.15 to 652.95	>0.05
16 days vs. 14 days	309.766	-230.79 to 850.32	>0.05

Table S2. Dry density and peak compressive strength of soil under compaction or self-weight consolidation at different moisture contents.

Initial moisture content (% dry weight)	Dry density (mg/mm^3)	Peak compressive strength (kPa)
15	1.62	941.19
20	1.57	544.11
30	1.74	1788.11
40	1.67	1021.87
50	1.48	338.19
60	1.40	243.78

Table S3. Tukey's HSD post-hoc test for peak compressive strength of soil under compaction or self-weight consolidation at different moisture contents ($\alpha = 0.05$). In column ‘Comparison’ the number on the right is compared to the number on the left. A positive sign in the ‘Mean diff.’ column suggests that the number to the left in the ‘Comparison’ column is greater than the number on the right.

Comparison	Mean diff.	95% CI of diff.	Summary
20% vs. 15%	-397.08	-894.12 to 99.96	>0.05
40% vs. 30%	-766.236	-1471.58 to -60.88	<0.05
50% vs. 30%	-1449.914	-2125.23 to -774.59	<0.001
60% vs. 30%	-1544.324	-2292.46 to -796.18	<0.001
50% vs. 40%	-683.678	-1359.00 to -8.35	<0.05
60% vs. 40%	-778.088	-1526.22 to -29.94	<0.05
60% vs. 50%	-94.409	-814.30 to 625.48	>0.05
